# Shared features in ear and kidney development – implications for oto-renal syndromes

**DOI:** 10.1242/dmm.050447

**Published:** 2024-02-14

**Authors:** Scarlet Xiaoyan Wang, Andrea Streit

**Affiliations:** Centre for Craniofacial and Regenerative Biology, King's College London, London SE1 9RT, UK

**Keywords:** Congenital anomalies, Ear, Embryo development, Kidney, Organogenesis

## Abstract

The association between ear and kidney anomalies has long been recognized. However, little is known about the underlying mechanisms. In the last two decades, embryonic development of the inner ear and kidney has been studied extensively. Here, we describe the developmental pathways shared between both organs with particular emphasis on the genes that regulate signalling cross talk and the specification of progenitor cells and specialised cell types. We relate this to the clinical features of oto-renal syndromes and explore links to developmental mechanisms.

## Teaser

**Summary:** This Review summarizes the developmental pathways shared between inner ear and kidney formation and explores the mechanisms underlying oto-renal syndromes.

## Introduction

The anatomy and function of the ear and kidney differ considerably (Box 1), as does their embryonic development. Nevertheless, renal and ear anomalies are associated in humans (Blunston et al., 2015; Ho and Power, 2010; Karasawa and Steyger, 2015; Lin et al., 2016; Quick and Hoppe, 1975; Verdel et al., 2008). Over 30 complex syndromes have been described that affect both inner ear and kidney function (Tables 1 and 2), including Alport syndrome and branchio-oto-renal (BOR) syndrome (Izzedine et al., 2004; Kohelet and Arbel, 2000; Leung and Robson, 1992). Hearing loss is also associated with low estimated glomerular filtration rate (see Glossary, Box 2) and late chronic kidney disease (Seo et al., 2015). Furthermore, small molecules, such as loop diuretics (Box 2), chemotherapy drugs and aminoglycosides, cause both ototoxicity (Box 2) and nephrotoxicity (Box 2) (Ding et al., 2016; Humes, 1999; Jiang et al., 2017). Despite their difference in embryonic origin, the ear and kidney share some developmental pathways and, in adults, they share physiological processes required for normal function. These similarities likely explain the association between ear and kidney disease.

**Box 1. Anatomy and function of the inner ear and kidney**

The inner ear and kidney are anatomically complex organs with multiple specialised cell types, of which correct spatial arrangement is critical for organ function. The inner ear is the sensory system responsible for hearing and balance (Alsina and Whitfield, 2017; Barald and Kelley, 2004; Driver and Kelley, 2020; Magariños et al., 2012), whereas the kidney maintains the homeostasis of body fluids and electrolytes via filtration, reabsorption and secretion of solutes and hormones (Bovee, 1986; Dressler, 2006, 2009). Their anatomy is very different, although they share several features in fluid management mediated by, for example, ion channels, transporters and gap junctions.

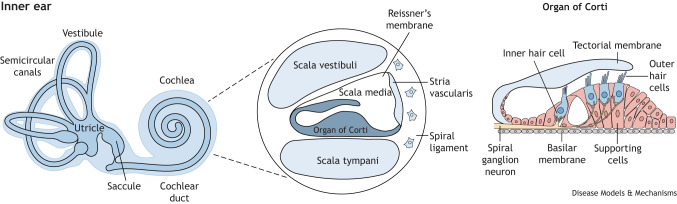

**Fig. 1.**
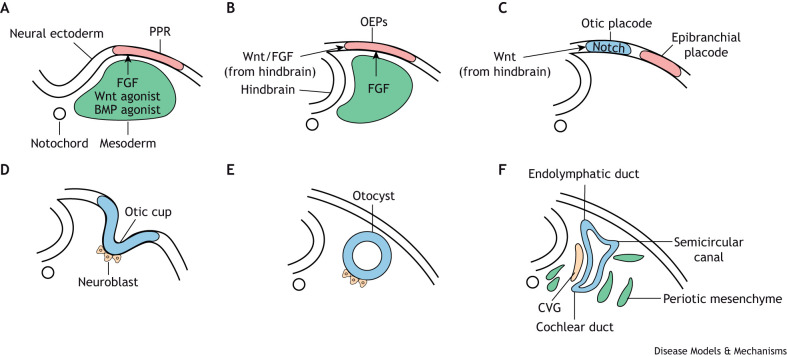
**Inner ear development.** (A) At early neurulation, mesoderm-derived fibroblast growth factors (FGFs) together with Wnt and BMP antagonists induce the pre-placodal region (PPR) in the ectoderm (Ahrens and Schlosser, 2005; Litsiou et al., 2005). (B) FGFs from the mesoderm and hindbrain and Wnts from the hindbrain specify otic-epibranchial progenitors (OEPs). (C) OEPs segregate into the otic and epibranchial placodes. Otic placode formation requires high Wnt and low FGF signalling (Freter et al., 2008; Ohyama et al., 2006); within the otic placode, Notch signalling cooperates with Wnt (Jayasena et al., 2008). (D) Around E9-E10 in mice, the otic placode invaginates to form the otic cup while neuroblasts delaminate. (E) The otic cup separates from the surface ectoderm to form the otocyst. (F) The semicircular canals and the endolymphatic duct begin to emerge dorsally, whereas the cochlear duct grows out ventrally. Neuronal precursors continue to delaminate and coalesce to form the cochleovestibular ganglion (CVG).

The adult human inner ear is composed of bony and membranous labyrinths. The former is a cavity located within the petrous part of the temporal bone within the cranium (Glueckert et al., 2018) and is divided into three parts: the cochlea, the vestibule and the semicircular canals. The membranous labyrinth is encased in the bony labyrinth and consists of three corresponding structures: the three semicircular ducts, the vestibule, including the utricle and saccule, and the cochlear duct (Fig. 1). The semicircular canals are oriented orthogonally to one another to mediate balance in all three planes of movement, and the vestibule is responsible for the detection of linear and gravitational acceleration. The cochlear duct is responsible for auditory perception and comprises three interconnected fluid-filled compartments: the scala vestibuli, the scala media and the scala tympani. The scala vestibuli and scala tympany are located in the upper and lower compartments of the cochlear duct, respectively, and are filled with perilymph (Box 2) enriched in sodium ions (Na^+^). The central compartment, the scala media, is filled with endolymph (Box 2) high in potassium ions (K^+^). It is separated from the other two scalae by tight and leaky barriers located in the basilar membrane and in Reissner's membrane, composed of different highly specialized epithelial cells (Salt and Plontke, 2018).

Each part of the membranous labyrinth houses a specialised sensory epithelium: the organ of Corti in the scala media, the maculae in the utricle and saccule, and the cristae at the base of the semicircular canals. These epithelia are arranged in a regular mosaic, with mechanosensory hair cells associated with one or more layers of non-sensory supporting cells. In the organ of Corti, there are typically three rows of outer hair cells, and a single row of inner hair cells. Each hair cell contains a cluster of modified microvilli, called stereocilia, and their deflection in response to movement leads to K^+^ influx and depolarization of the cell membrane. This triggers the release of neurotransmitters at their basal surface, which induces action potentials in the innervating auditory neurons (Nordang et al., 2000; Oestreicher et al., 2002). The lateral wall of the cochlear duct, composed of the spiral ligament and the stria vascularis, is essential to create and maintain the K^+^ equilibrium potential (Bovee, 1986). This, therefore, contributes to the driving force of sensory transduction (Marcus et al., 1985; Takeuchi et al., 2000).

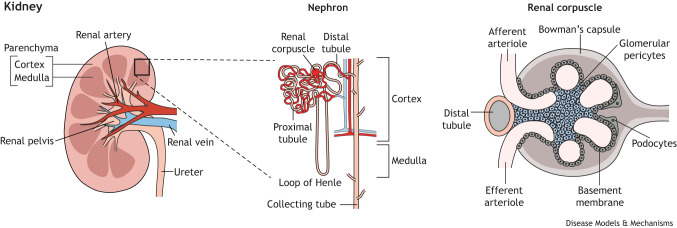

The paired bean-shaped kidneys are the central organs to maintain homeostasis of the human body systems. Their two functional sections, the renal parenchyma and the renal pelvis, are managed and develop independently. The renal parenchyma includes the renal cortex and the renal medulla with the key structural unit of the kidney, the nephron, being located within the parenchyma. The nephron filters the blood to remove waste and reabsorb water and nutrients to maintain homeostasis. There are approximately 15,000 nephrons per kidney in mice and one million in humans (Bertram et al., 2011; Takasato and Little, 2015). Each nephron connects to the collecting tube network through which the urinary filtrate passes to the renal pelvis, the upper end of the ureter, and then exits the kidney leading to the bladder.

The nephron is composed of the renal corpuscle, the proximal tubule, the loop of Henle, the distal tubule and the associated capillary network (the arterioles and renal artery are shown in red and the renal vein is shown in blue), similar to stria vascularis in the cochlea. The renal corpuscle, consisting of the glomerulus (the convoluted knot of leaky blood vessels) and Bowman's capsule (or glomerular capsule), is located at the proximal part of the nephron, within the cortex. Renal filtration starts from the glomerulus (Scott and Quaggin, 2015), which is supported by closely associated pericytes (Box 2). The filtered fluid flows into the interstitial space of the Bowman's capsule, which is lined by podocytes (Box 2). Together with the glomerular endothelial cells, these highly specialized podocytes form the glomerular basement membrane (Brunskill et al., 2011), consisting of nine proteins that are also expressed in the cochlear basement membrane (Liu et al., 2015; Meyer zum Gottesberge and Felix, 2005; Miner, 2011; Zehnder et al., 2005). Acting as a filtration barrier, the glomerular basement membrane only allows the passage of water and small solutes into the nephron, while preventing the entry of solutes with larger molecular mass, such as albumin and immunoglobulins.

The proximal tubule emerges from the renal corpuscle, and the epithelial membrane of the proximal tubule contains various channels and transporters that play a key role in molecular transport across the membrane (Brunskill et al., 2008; Thiagarajan et al., 2011). This mediates the reabsorption of about 70% of NaCl, glucose, amino acids and essential minerals from the ultrafiltrate (Box 2) to the surrounding vascular capillaries, whereas K^+^ channels are involved in regulating cell volume and maintaining membrane potential during depolarizing Na^+^-coupled transport. The role of these K^+^ channels, which function similarly in the loop of Henle, distal tubule and collecting tube, resembles their role in sensory transduction in the cochlea (Zdebik et al., 2009). The loop of Henle, which connects the proximal tubule to the distal convoluted tubule, plays a key role in urine concentration by reabsorbing NaCl and K^+^ into the ultrafiltrate (de Rouffignac, 1972). The distal tubule also plays a critical role in the homeostasis of multiple ions, including Na^+^ and Ca^2+^, and participates in water uptake and pH balance (Subramanya and Ellison, 2014). Overall, the distal tubule and collecting tube, where the ultrafiltrate funnels into, fine tune renal acid, fluid volume and electrolyte excretion, whereas the cells in the glomerular apparatus monitor salt levels and regulate fluid flow into the glomerulus, and thus systemic blood pressure (Bell et al., 2003; Grassi et al., 2015).

**Box 2. Glossary**

**Ampullae:** a bulbous expansion at the base of each semicircular canal housing the sensory epithelium containing the hair cells.

**Cochlear duct:** endolymph-filled cavity inside the cochlea.

**Conductive hearing loss:** hearing loss due to defects in the outer and middle ear that prevents sound to travel.

**Craniofacial microsomia:** craniofacial abnormality in which one part of the face is smaller than normal.

**Craniosynostosis:** a congenital abnormality in which skull bones fuse too early.

**Decorin:** a protein encoded by the *DCN* gene in humans. It regulates many pathological processes of cancer.

**Endolymph:** physiological fluid in the membranous labyrinth of the inner ear.

**FAT4:** a protein encoded by the *FAT4* gene in humans. It functions as a Hippo signalling regulator.

**Glomerular filtration rate:** the volume of fluid filtered from the kidney glomerular capillaries into the Bowman's capsule per unit time. It describes the flow rate of filtered fluid through the kidney.

**Hippo pathway:** an evolutionarily conserved serine/threonine kinase signalling pathway that controls organ size by regulating cell proliferation, apoptosis and stem cell self-renewal.

**Hydronephrosis:** a condition where one or both kidneys become swollen as the result of a build-up of urine.

**Loop diuretics:** medication that acts on the Na-K-Cl cotransporter along the thick ascending limb of the loop of Henle in nephrons of the kidneys.

**Nephrotoxicity:** toxic effects on the kidney due to medication.

**Nucleosome remodelling and deacetylase (NuRD) complex:** a major chromatin remodelling complex that plays an important role in regulating gene transcription, genome integrity and cell cycle progression.

**Otic placode:** a transient thickening of the surface ectoderm located adjacent to the hindbrain that is a precursor of the inner ear.

**Ototoxicity:** toxic effects on the inner ear due to medication.

**Pericytes:** multi-functional cells of the microcirculation that wrap around endothelial cells lining capillaries.

**Perilymph:** extracellular fluid in the inner ear that fills the space within the bony labyrinth surrounding the membranous labyrinth.

**Periotic mesenchyme:** mesenchyme surrounding the ear.

**Pillar cells, Hensen's cells and Deiters' cells:** supporting cell types in the sensory epithelium of the cochlea.

**Podocytes:** highly specialized epithelial cells in the Bowman's capsule that wrap around capillaries of the glomerulus.

**Primitive streak:** site of gastrulation in amniote embryos.

**Renal agenesis:** a complete absence of one (unilateral) or both (bilateral) kidneys.

**Renal aplasia:** kidney that has failed to develop beyond its most primitive form.

**Saccule:** similar to the utricle, the saccule is a membranous sac containing a patch of sensory hair cells that detect linear acceleration and head tilts.

**Semicircular canals:** three interconnecting canals that are part of the bony labyrinth of the inner ear. They are filled with perilymph and contain the membranous labyrinth, which in turn provide information on angular acceleration.

**Sensorineural hearing loss:** hearing loss due to damage of the cochlear hair cells, auditory nerve or central nervous system.

**Synostosis:** early fusion of two or more bones.

**Ultrafiltrate:** the fluid that has passed across a semipermeable membrane (a membrane that allows some substances to pass through but not others) due to a driving pressure. In the kidney, it occurs at the barrier between the blood and the filtrate in the Bowman's capsule.

**Utricle:** similar to the saccule, the utricle is a membranous sac containing a patch of sensory hair cells that detect linear acceleration and head tilts.

**Vesicoureteral reflux:** a condition in which urine flows backward from the bladder to one or both ureters and sometimes to the kidneys.

**Table 1.**
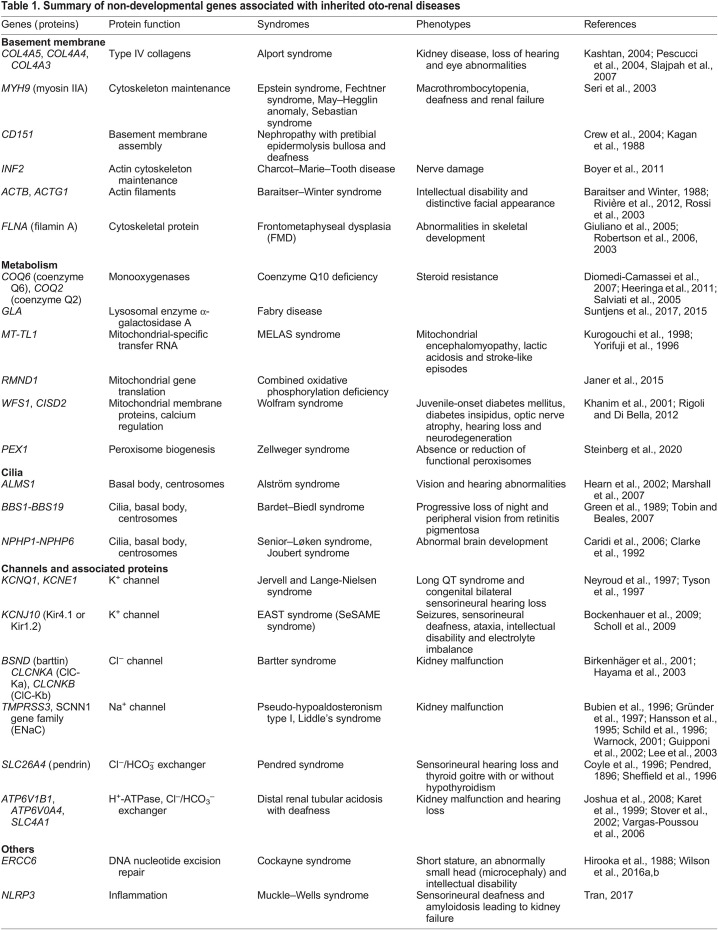
Summary of non-developmental genes associated with inherited oto-renal diseases

**Table 2.**
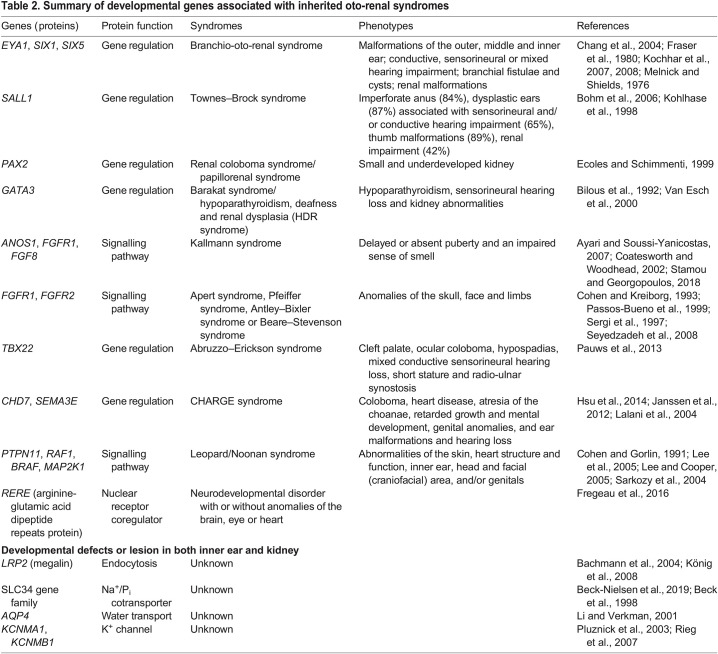
Summary of developmental genes associated with inherited oto-renal syndromes

Inherited oto-renal diseases are due to mutations in genes belonging to two general categories, 36 of which are expressed in the cochlea (Box 1) and the kidney (Liu et al., 2004). The first group includes genes that encode common specialized proteins, such as structural (e.g. basement membrane), metabolic/mitochondrial, ciliary, ion channel and transporter proteins (see Table 1 for associated pathologies). Comprehensive reviews of this group of genes are found elsewhere (Izzedine et al., 2004; Lang et al., 2007; Peters et al., 2004; Phelan and Rheault, 2018). The second group includes many developmental genes that control the formation of the inner ear and kidney (Table 2). In the last two decades, their early development has been characterized in detail and many of the molecular mechanisms coordinating their assembly, patterning and morphogenesis have been identified. However, the developmental mechanisms shared between both organs have not been explored and are less understood. Therefore, these shared developmental mechanisms will be the focus of this Review. First, we will briefly describe early development of the inner ear and kidney, and then discuss how multiple signalling pathways are used repeatedly in both organs and how cell-specific transcription factors control cell identity and tissue morphogenesis. Together, these shed light on the developmental connections between the inner ear and kidney and provide insight into the molecular mechanisms underlying oto-renal syndromes.

## An overview of early inner ear and kidney development

In the embryo, inner ear and kidney primordia arise from different germ layers, form at different times and at different speeds. Here, we provide a brief overview of their early development, before examining their shared developmental mechanisms more closely in the context of oto-renal syndromes. Extensive reviews of ear and kidney development can be found elsewhere (Barald and Kelley, 2004; Bok et al., 2007; Dressler, 2006, 2009; Driver and Kelley, 2020; Fekete and Wu, 2002; O'Brien et al., 2016; Sai and Ladher, 2015; Takasato and Little, 2015; Wu and Kelley, 2012).

Ear development begins with the induction of the otic placode (Box 2), followed by formation and patterning of the otocyst (Fig. 1A-E). These processes occur between embryonic day (E) 8.5 and E11.5 in mice and approximately between Carnegie stage (CS) 9 and CS19 in humans. Extrinsic signals induce regionally restricted expression of transcription factors in the otocyst (Bok et al., 2005, 2011; Giraldez, 1998; Morsli et al., 1998; Wu and Oh, 1996). These define different functional territories, such as neurosensory versus non-neurosensory domains, and future cochlea versus the vestibular apparatus and endolymphatic duct (Fig. 1F) (Brigande et al., 2000; Fekete and Wu, 2002). Simultaneously, cell type specification occurs. For example, the neural-sensory competent domain first generates neuronal precursors, which subsequently delaminate and coalesce, forming the cochleovestibular ganglion. Later, neural-sensory competent cells give rise to prosensory cells that will generate hair cells (HCs) and supporting cells (SCs) afterwards. Likewise, cells that form the cochlea and saccule (Box 2) are set aside in the ventral otocyst, whereas semicircular canals (Box 2) and the utricle (Box 2) are specified dorsally (Bok et al., 2007; Morsli et al., 1998). Thereafter, complex morphogenetic events accompanied by differential proliferation, cell death, cell shape changes and reorganisation transform the otocyst into the complex structure of the adult ear (Haugas et al., 2010; Lang et al., 2000; Montcouquiol et al., 2003; Pirvola et al., 2002, 2004). The signalling and transcriptional events that control these morphogenetic events remain poorly understood.

**Fig. 2.**
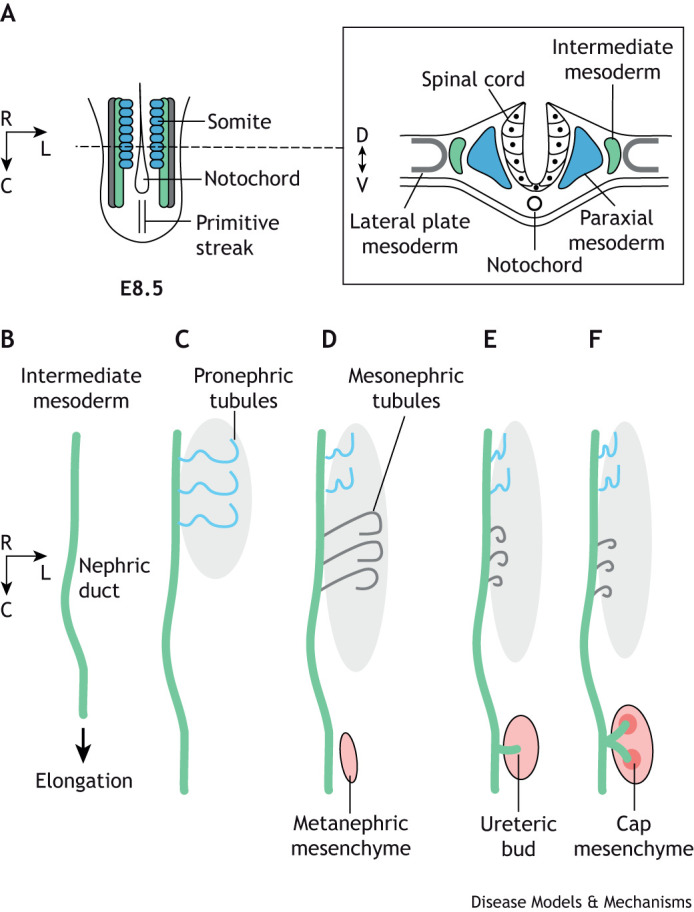
**Amniote kidney development.** (A) Schematic of the tail end of a mouse E8.5 embryo showing the remnants of the primitive streak (Box 2). The intermediate mesoderm (green) is patterned along the medio-lateral axis and requires intermediate levels of BMP and nodal signalling (James and Schultheiss, 2003; James and Schultheiss, 2005; Obara-Ishihara, 1999). The kidney arises from the intermediate mesoderm. Cross-section at approximately the 6th somite level (dotted line) is shown on the right. (B-F) Temporal and spatial order of kidney formation. (B) The nephric (or Wolffian) duct grows caudally by proliferation and extension at E8.5 [∼Carnegie stage (CS) 10 in humans]. (C) Pronephric tubules develop at the rostral end at E9.0. (D) Mesonephric tubules form posterior to the pronephros in the mid-thoracic region at E10; cells posterior and adjacent to the nephric duct aggregate to form the metanephric mesenchyme (pink). (E) By E10.5 (∼CS12 in humans), the pronephros and mesonephros degenerate and the ureteric bud emerges from the nephric duct and invades the metanephric mesenchyme. (F) By E11.5, the ureteric bud has bifurcated and induces the cap mesenchyme surrounding its tips, which will later develop into nephrons. C, caudal; L, lateral; R, rostral.

The kidney develops from the intermediate mesoderm in a spatial-temporal sequence regulated by both medio-lateral and rostro-caudal patterning signals (Fig. 2) (McMahon, 2016; Saxen, 1987). Pronephros and mesonephros arise anteriorly, whereas the metanephros, later forming the adult kidney, forms posteriorly. Regional specification of the intermediate mesoderm is regulated by Hox genes together with inducing signals from surrounding tissue. Hox4 group genes are important for the specification of the anterior intermediate mesoderm (Preger-Ben Noon et al., 2009), whereas Hox11 genes are critical for the formation of the posterior metanephric kidney, including the metanephric mesenchyme and the ureteric bud (Takasato and Little, 2015). Reciprocal signalling between these two tissues promotes ureteric bud branching (Chi et al., 2009; Dudley et al., 1995; Luo et al., 1995; Michos et al., 2007; Miyazaki et al., 2000). The invading bud triggers the condensation of cap mesenchyme cells around the bud tips (Carroll et al., 2005), inducing the mesenchymal-epithelial transition, followed by polarization of the epithelium and nephrogenesis. The cap mesenchyme cells are nephron progenitors and their maintenance is critical for continuous generation of nephrons. They are surrounded by stromal progenitors and cells that generate the vascular elements of the kidney, including the capillaries of the glomeruli that later form the mature glomerulus (Little and McMahon, 2012).

## Shared developmental pathways and related syndromes

Inner ear and kidney development involve elaborate processes orchestrated by dynamic signalling and gene expression. Given this complexity, it is not surprising that disruptions in these pathways lead to kidney and/or hearing dysfunction. In certain oto-renal syndromes, ear and kidney anomalies occur at early developmental stages (Table 2), suggesting that similar but not identical gene networks control the formation of both organs. Here, we provide an overview of shared developmental pathways that have been implicated in oto-renal syndromes.

## The PAX-EYA-SIX network

The PAX-EYA*-*SIX regulatory cassette is evolutionarily conserved from flies to vertebrates. In vertebrates, these genes play key roles during the development of different organs, including the inner ear and kidney. Although SIX and PAX family members are transcription factors, EYA factors are transcriptional co-activators that interact with SIX proteins, although they also function independently as tyrosine phosphatases (Li et al., 2003; Torres et al., 1996; Xiong et al., 2009; Xu et al., 2014a,b, 1999, 2003; Zheng et al., 2003). Their function has mainly been investigated in mice with loss of *Six1*, *Eya1* or *Pax2* function leading to inner ear and kidney defects with similar features*.* Pathogenic mutations in this network have been identified in patients with BOR syndrome and renal coloboma syndrome, highlighting its role in human ear and kidney development.

### Multiple roles of *Six1* and *Eya1* in ear and kidney development and their relation to BOR syndrome

Genetic analysis reveals that mutations in *EYA1* (Buller et al., 2001; Chang et al., 2004; Johnson et al., 1999; Kalatzis et al., 1998; Li et al., 2018; Musharraf et al., 2014; Orten et al., 2008; Wang et al., 2018), *SIX1* (Kochhar et al., 2008; Ruf et al., 2004; Shah et al., 2020) and *SIX5* (Hoskins et al., 2007) cause BOR syndrome, an autosomal dominant developmental defect characterised by branchial and external ear malformations, hearing loss and renal anomalies (Abdelhak et al., 1997; Fraser et al., 1980; Sanggaard et al., 2007). The major feature of BOR syndrome is hearing loss (in 93% of patients), which can be conductive (30%; Box 2), sensorineural (20%; Box 2) or mixed (50%) and varies in age of onset (Chen et al., 1995). 67% of patients present with renal defects, ranging from mild hypoplasia or dysplasia to renal agenesis (Box 2), ureteropelvic junction obstruction and vesicoureteral reflux (Box 2) (Chang et al., 2004). The incidence of BOR syndrome is 1 in 40,000 and accounts for about 2% of profoundly deaf children (Fraser et al., 1980). Approximately 40% of patients with BOR syndrome carry a pathogenic mutation in *EYA1* (Chang et al., 2004), whereas 9-10% have mutations in *SIX1* or *SIX5* (Kochhar et al., 2008; Song et al., 2013). For the remaining patients with BOR syndrome, causative mutations have not been identified. Mutations in the conserved EYA domain abrogate its interaction with SIX1/SIX2 proteins, leading to rapid EYA1 degradation and/or altered target gene expression (Buller et al., 2001; Li et al., 2018; Musharraf et al., 2014; Sanggaard et al., 2007). Mutations in *Six1*/*Six5* are frequently found in the DNA-binding homeodomain or in the SIX domain responsible for protein-protein interactions. Mutations in the latter disrupt SIX and EYA1 interaction, whereas mutations in the former interfere with SIX-DNA binding, both of which are vital for normal SIX1/SIX5 function (Hoskins et al., 2007; Kochhar et al., 2008; Mehdizadeh et al., 2021; Ozaki et al., 2002; Ruf et al., 2004; Shah et al., 2020). Thus, mutations identified in patients with BOR syndrome are likely to interfere with the diverse functions of SIX and EYA proteins during ear and kidney formation, and further investigation of the molecular mechanisms will enhance our understanding of BOR syndrome pathogenesis.

During early ear development, *Eya1* (Bane et al., 2005; Kalatzis et al., 1998; Sahly et al., 1999) and *Six1* (Bricaud and Collazo, 2006; Pandur and Moody, 2000; Zheng et al., 2003) are co-expressed in the otic placode together with *Pax2* (Christophorou et al., 2009; Hutson et al., 1999; Torres et al., 1996), but are also found in the periotic mesenchyme (Box 2). In mice, *Eya1* and *Six1* expression becomes restricted to the ventral otocyst around E9.5, where auditory and vestibular sensory epithelia form later. At E11.5, they are expressed in the cochlear duct (Box 2), and throughout the thickness of all sensory epithelia at E13.5. By E15.5, their expression is progressively restricted to differentiating HCs in the cochlear and the vestibular sensory epithelium (Ozaki et al., 2004; Zou et al., 2008, 2006).

Using a dominant repressor form of *SIX1*, experiments in chicks revealed that activation of SIX1 target genes is required to form a morphological placode and to express otic markers such as *PAX2* (Christophorou et al., 2009), suggesting that SIX1 plays a role in ear progenitor formation. In mice, loss of *Six1* or *Eya1* does not affect initial ear development, as the otocyst forms in the correct position in single-knockout or double-heterozygote mice (Zheng et al., 2003; Zou et al., 2008). However, normal growth and patterning of the otocyst is severely affected and ear development is arrested (Xu et al., 1999). At E9.5, *Six1*- or *Eya1*-deficient embryos show reduced proliferation and increased apoptosis in the otic epithelium, whereas in *Eya1*/*Six1* double-knockout embryos, the phenotype is exacerbated (Ozaki et al., 2004; Zheng et al., 2003; Zou et al., 2006)*.* As a result, otocysts are smaller in mutant mice compared to those in wild-type littermates and the expression of several otic markers is abolished or shifted (Zheng et al., 2003; Zou et al., 2006). Indeed, *Six1* and *Eya1* play a general role in cell proliferation and survival (Li et al., 2003).

As described above, *Six1* and *Eya1* are initially expressed in progenitors throughout all sensory epithelia and then become restricted to differentiating HCs. They, therefore, may control formation of the prosensory domain and later HC development (Bricaud and Collazo, 2006; Zhang et al., 2017; Zheng et al., 2003). Deletion of *Six1* at E11.5 using the *Eya1*^CreER^ or *Sox2*^CreER^ inducible systems causes defects in progenitor cell proliferation and elongation of the prosensory primordium, leading to smaller cochlear ducts, as well as smaller utricular and saccular maculae at E14.5 (Zhang et al., 2017). By E18.5, these mice have fewer HCs, abnormal cell morphology and defective patterning of the sensory epithelium (Zhang et al., 2017). *Eya1* regulates sensory development in a concentration-dependent manner, with progressive depletion resulting in increasingly severe malformations. An allelic series combining the wild-type, hypomorphic (*Eya1*^bor^) and null (knockout allele, *Eya1*^−^) alleles of Eya1 revealed that a 79% reduction in EYA1 protein level prevents cochlear and vestibular sensory epithelia formation, whereas a 27.5% reduction of EYA1 shows no phenotype (Zou et al., 2008). Animals with 21-49% of normal EYA1 levels display a shortened cochlea and fewer differentiated HCs at E18.5, with disorganised HCs and short or disoriented stereocilia bundles (Zou et al., 2008). Thus, the formation and size of inner ear sensory epithelia depend on the function of SIX1 and EYA1 at specific developmental stages, as does the differentiation of HCs. In agreement with this, misexpression of SIX1 and EYA1 in the mouse cochlea induces ectopic HC formation (Ahmed et al., 2012a).

*Six1* and *Eya1* also play a role in inner ear neurogenesis. In *Xenopus*, low levels of SIX1 promote proliferation of neuronal progenitors by controlling *Sox2* expression, whereas high levels allow the activation of neurogenic genes (Schlosser et al., 2008). In mice, although not required for neuroblast specification, *Eya1* and *Six1* are essential for maintaining neuroblast survival, differentiation and/or maturation. Loss of either factor results in abnormal cell death and neuroblast degeneration from E9.5 onwards, ultimately leading to cochlear-vestibular ganglion loss (Friedman et al., 2005; Zou et al., 2004). In *Eya1* and *Six1* double mutants, neuroblasts are virtually absent (Ahmed et al., 2012b; Zheng et al., 2003). In contrast, overexpression of *Eya1* and *Six1* in the otocyst (E9.25-E9.5) and in the non-sensory epithelial cells of the cochlea (E13.5-E14.0) induces ectopic neurons (Ahmed et al., 2012b).

Taken together, these findings show that SIX1 and EYA1 act in a context-dependent manner, likely through activating cell-specific targets and cooperating with different cofactors. SIX1-bound enhancer regions were recently identified in mouse cochlear epithelia (Li et al., 2020). At E13.5, SIX1 appears to control several early ear-specific transcription factors, which themselves have important roles in ear development, including *Six1* itself, *Six2*, *Sox2* and *Pax2*. At E16.5, the key regulators for HC fate, namely *Atoh1*, *Pou4f3* and *Gfi1*, are regulated by SIX1, as are hair-bundle regulators. In this context, SIX1 cooperates with RFX proteins to promote HC formation (Li et al., 2020). Although this study did not investigate neurons, functional experiments revealed key differences in SIX1 and EYA1 function in neurons and HCs. To promote neurogenesis, EYA1 and SIX1 interact with BRG1 (or SMARCA4) and BAF170 (SMARCC2), components of the SWI/SNF chromatin-remodelling complex (Ahmed et al., 2012b), and cooperate with SOX2, which in turn antagonises HC differentiation (Ahmed et al., 2012a,b).

Finally, SIX1 and EYA1 also control signalling molecules and pathway components that are critical for normal ear formation. Several appear to be directly regulated by SIX1 (Li et al., 2020). Analysis of *Six1*- and *Eya1*-mutant mice showed that both factors are required for the activation of *Fgf3* and the maintenance of *Fgf10* expression in the otocyst, and of *Fgf8* in inner HCs (IHCs) (Xu et al., 1999; Zhang et al., 2017; Zheng et al., 2003; Zou et al., 2006). The importance of the FGF pathway is discussed in a later section. In summary, SIX1 and EYA1 form a transcription factor complex that is crucial at different stages in inner ear development for the maintenance of otic placode, neuronal and neurosensory progenitor proliferation, growth of the sensory epithelia and, consequently, morphogenesis of the cochlea, specification and differentiation of HCs, and sensory neurogenesis.

In the mouse kidney, *Eya1* is co-expressed with two Six family members, *Six1* and *Six2*, in the metanephric mesenchyme from E10.5 onwards (Oliver et al., 1995; Sajithlal et al., 2005). In human embryos, these cells co-express *EYA1* and *SIX1* (gestation week 16-22) (Li et al., 2002). *Eya1*- or *Six1*-deficient mice lack metanephric kidneys; the ureteric buds do not grow out and metanephric induction does not occur (Laclef et al., 2003; Xu et al., 1999). *Six1* and *Six2* expression depends on *Eya1*, with SIX1 also regulating *Six2* expression (Xu et al., 1999, 2003). Therefore, *Eya1* lies at the top of the genetic hierarchy controlling metanephric mesenchyme specification.

Within the metanephric mesenchyme, the EYA-SIX cassette plays a dual role, controlling signalling between the mesenchyme and the ureteric bud, and regulating the progenitor state of metanephric mesenchyme cells themselves. In the absence of *Eya1* function, the metanephric mesenchyme is not specified and the ureteric bud does not form (Sajithlal et al., 2005; Xu et al., 1999). Like in the inner ear, *Eya1* affects renal development in a dosage-dependent manner. As little as 20% of normal EYA1 levels are sufficient to establish the metanephric mesenchyme and induce ureteric bud formation, but this is not sufficient for normal branching (Sajithlal et al., 2005). Molecularly, EYA1 forms a complex with HOX11 paralogues and PAX2 to activate the expression of *Six2*, which in turn activates *Gdnf* expression by binding to its promoter to support ureteric bud outgrowth and invasion (Brodbeck et al., 2004; Gong et al., 2007). Mice deficient in *Eya1*, *Hox11* or *Pax2*, as well as *Six1*, show a loss or reduction of *Six2* and *Gdnf* expression (Brophy et al., 2001; Wellik et al., 2002; Xu et al., 1999). In *Six1*-deficient mice, although the ureteric bud starts to grow out, it fails to invade the mesenchyme and branching is impaired (Xu et al., 2003). Thus, EYA1 acts in combination with HOX11, PAX2 and SIX1/SIX2 to promote ureteric bud outgrowth and branching by regulating *Gdnf* expression (Nie et al., 2011; Sajithlal et al., 2005).

*Six1*, *Six2* and *Eya1* also regulate the balance between proliferation and differentiation in the metanephric mesenchyme (Gong et al., 2007; Xu et al., 2014b). Conditional inactivation of *Eya1* in the cap mesenchyme leads to *Six2* downregulation. In turn, *Six2* is required for maintaining the nephron progenitor pool; *Six2* inactivation leads to premature differentiation of progenitors into nephrons and to increased apoptosis, ultimately resulting in the loss of the progenitor pool (Combes et al., 2019a; Kobayashi et al., 2008; Self et al., 2006; Xu et al., 2014a,b). Likewise, in the absence of *Six1* function, apoptosis is increased in the metanephric mesenchyme (Xu et al., 2003). Interestingly, forced expression of *Six1* in nephron progenitors inhibits premature epithelialization of these progenitors in *Six2*-knockout mice, but fails to rescue the proliferation defects and cell death (Xu et al., 2022). These observations indicate that although SIX1 and SIX2 share some functions, they are not completely interchangeable. They also suggest that EYA1 and SIX proteins regulate the balance of self-renewal and differentiation of nephron progenitor cells. Molecularly, SIX2 and EYA1 function by interacting with BRG1 to regulate key target genes (Li et al., 2021), similar to what is observed in ear neurogenesis (Ahmed et al., 2012b).

Recent studies have identified both SIX1 and SIX2 targets in mouse and human nephron progenitors, defining shared and unique functions of both family members, as well as human-specific SIX1/SIX2 targets (O'Brien et al., 2016). In humans, *SIX1* is a SIX2 target, and both SIX1/SIX2 activate *SIX2* and bind putative enhancers around *SIX1* (O'Brien et al., 2016). This autoregulatory feedback loop drives continued *SIX1*/*SIX2* expression during active nephrogenesis. In contrast, in mice, SIX2 binds to the *Six2* enhancer but not to enhancers that control *Six1* (Xu et al., 2022). These data suggest that, although SIX1/SIX2 autoregulation is conserved, their upstream regulation diverges in mouse and human nephron progenitors, which might contribute to species differences in the duration of nephrogenesis and the final nephron numbers. In addition, these studies have identified many *SIX1*/*Six1* and *SIX2*/*Six2* targets, including *SALL1* and *EYA1*/*EYA4*, genes involved in cell cycle regulation, as well as members of various signalling pathways critical for the crosstalk between different cell populations in the developing kidney.

In summary, SIX family members and EYA1 are important for different steps in ear and kidney development, acting in a context-specific manner. They are critical for the specification and maintenance of progenitor populations and for the generation specialised cell types. In otic neurons and nephron progenitors, they control the balance between proliferation and differentiation. During cell fate specification, SIX1 and EYA1 cooperate with tissue-specific cofactors and interact with different chromatin-remodelling complexes to control target gene expression. In humans, mutations in *SIX1*/*SIX2* and *EYA1* are likely to affect downstream events, which contribute to various phenotypes of BOR syndrome.

### The role of PAX2 in ear and kidney formation and associated renal coloboma syndromes

In humans, *PAX2* mutations cause renal coloboma syndrome, an autosomal dominant condition characterized by kidney abnormalities, optic nerve colobomas and hearing loss (Ecoles and Schimmenti, 1999). *PAX2* mutations can be identified in nearly half of the patients presenting with renal coloboma features (Schimmenti, 2011), whereas the underlying cause in the remaining half needs to be determined. A single-allele mutation in *PAX2* leads to kidney abnormalities in which they are small and abnormally formed (renal hypoplasia), with up to one-third of the patients developing end-stage renal disease. In addition, patients exhibit ocular defects (Favor et al., 1996; Porteous et al., 2000). Hearing loss is a variable feature of the syndrome, with up to 10% of affected individuals presenting with high-frequency hearing loss already in childhood (Nishimoto et al., 2001; Sanyanusin et al., 1995a,b; Schimmenti et al., 1997).

*Pax2* is among the earliest genes expressed in the otic placode and continues to be expressed throughout inner ear development (Hidalgo-Sánchez et al., 2000; Lawoko-Kerali et al., 2002; Nornes et al., 1990; Terzic et al., 1998; Torres et al., 1996). Likewise, it is widely expressed during the development of both the ductal and mesenchymal components of the urogenital system (Dressler et al., 1993; Winyard et al., 1996).

In zebrafish, *pax2a* and *pax8* promote otic fate: loss of either gene impairs ear development at early stages (Hans et al., 2004; Mackereth et al., 2005; McCarroll et al., 2012). Likewise, in chicks, *PAX2* coordinates otic fate, proliferation and placode morphogenesis (Christophorou et al., 2010; Freter et al., 2012). When *PAX2* expression is reduced in chick otic progenitors, the expression of some otic markers is lost, and cells fail to proliferate normally and elongate into a typical placode shape. As in fish, *Pax2* and *Pax8* also cooperate in the mouse inner ear. *Pax8* is expressed at early placode stages, followed by *Pax2* expression slightly later. In *Pax8-*null mice, the ear develops normally, despite some later hearing loss, whereas *Pax2* deletion leads to reduced outgrowth of the cochlear duct and defects in spiral ganglion formation (Bouchard et al., 2010; Burton et al., 2004; Torres et al., 1996). In *Pax2*/*Pax8* double-null mice, the ear does not develop past the otocyst stage (Bouchard et al., 2010). Together, these findings suggest that both factors have partially redundant functions, with *Pax2* being able to partially compensate for *Pax8*, but not vice versa.

At later stages, *Pax2* regulates the morphogenesis of both the auditory and vestibular systems via interaction with *Eya1*. *Pax2* and *Eya1* double-heterozygous-mutant mice displayed small or morphologically unidentifiable ampullae (Box 2) and shortened cochlea, whereas such defects were not observed in single-heterozygous mutants (Zou et al., 2006). Thus, similar to SIX1 and EYA1, PAX2 plays multiple roles in ear development.

During kidney development, *Pax2* is expressed in multiple compartments and *Pax2-*deficient newborn mice display a partially developed nephric duct and no metanephric development at all (Torres et al., 1995). *Pax2* also plays a role in maintaining the nephron progenitor pool in the metanephric mesenchyme by interacting with Hox11 paralogs and *Eya1* to activate expression of *Six2* and *Gdnf* (Brophy et al., 2001; Wellik et al., 2002). At the time of ureteric bud outgrowth and invasion into the metanephric mesenchyme, *Pax2* is highly expressed in the condensing mesenchyme, similar to *Eya1* and *Six1*/*Six2.* In *Pax2-*knockout mice, the mesenchyme-to-epithelium conversion to form nephrons is impaired: mesenchyme cells fail to aggregate and do not form epithelial cells (Dressler et al., 1993). When the mesenchyme-derived epithelium matures, *Pax2* expression is normally downregulated (Ryan et al., 1995; Winyard et al., 1996); therefore, persistent *Pax2* expression is associated with a variety of cystic and dysplastic renal diseases and correlates with restricted differentiation and continued proliferation of renal epithelial cells (Dressler et al., 1993; Dressler and Woolf, 1999; Eccles et al., 2002; Ostrom et al., 2000). In the foetal kidney, *Pax2* is also downregulated as cells leave the mitotic cycle (Eccles et al., 1995; Winyard et al., 1996). Like in ear progenitors, PAX2 appears to regulate proliferation versus differentiation in the metanephric mesenchyme, as well as its transition to epithelial cells.

PAX2 cooperates with PAX8 to specify nephric fate, like in the ear. In *Pax8*-deficient mice, the kidney develops normally (Mansouri et al., 1998), whereas *Pax2* and *Pax8* heterozygous embryos display hypoplastic kidneys. *Pax2* also interacts with *Eya1*, with double-heterozygous mice exhibiting abnormal ureteric bud branching morphogenesis (Sajithlal et al., 2005; Zou et al., 2006).

**Fig. 3.**
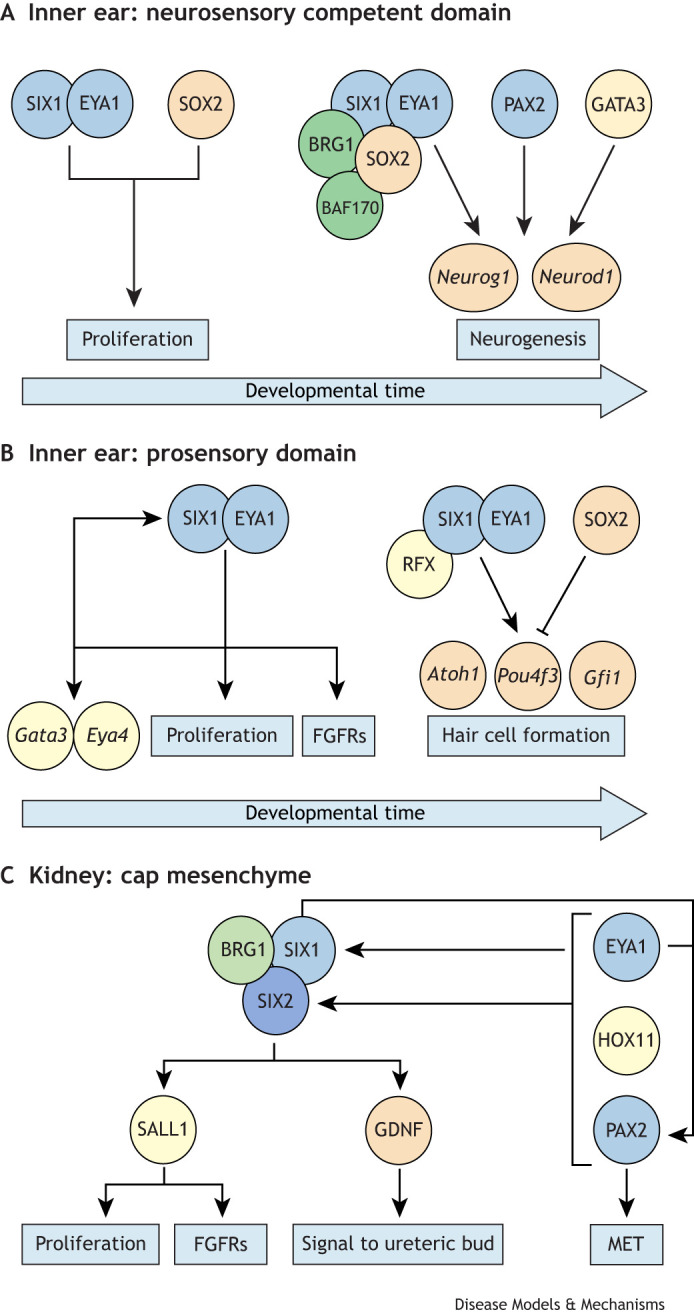
**Multiple roles of the SIX1-EYA1-PAX2 network in ear and kidney development.** (A) In the neurosensory competent domain of the inner ear, SIX1/EYA1 initially control proliferation together with SOX2. Later, they form a complex with chromatin remodellers BRG1 and BAF170 to activate neurogenic genes (*Neurog1* and *Neurod1*); PAX2 and GATA3 also promote neurogenesis. (B) In the prosensory domain of the inner ear, SIX1/EYA1 regulate themselves in a positive feedback loop promote proliferation and activate FGFRs as well as *Eya4* and *Gata3*. Later, SIX1 and EYA1 work in a complex with RFX proteins to promote hair cell formation via upregulation of *Atoh1*, *Pou4f3* and *Gfi1*, which is inhibited by SOX2. (C) In the cap mesenchyme, like in the ear, SIX1 works together with BRG1 to activate downstream targets, including SALL1, which in turn control proliferation and FGFRs. GDNF is under the control of SIX2 and signals to the adjacent ureteric bud. SIX1 and EYA1, in combination with HOX11 factors and PAX2, control *Six2*, as well as autoregulate themselves. SIX1 and EYA1 also control PAX2, which regulates the mesenchymal-epithelial transition (MET).

In summary, the PAX-EYA-SIX cassette plays multiple roles in both ear and kidney development (Fig. 3). This evolutionary conserved network was originally identified in the *Drosophila* eye, in which the *Pax6* orthologue (*ey*) lies at the top of the genetic hierarchy and is required to activate *Six1* and *Eya1* orthologues (*so* and *eya*, respectively) (Quiring et al., 1994; Ransom, 1979; Bonini et al., 1993). All three subsequently form a regulatory network that controls eye formation together with other cofactors. In vertebrates, the Six, Eya and Pax families have expanded, but the basic network was maintained and recruited into new tissues for new functions. However, the circuit architecture has changed in different contexts. In the metanephric mesenchyme, *Pax2* functions downstream of *Eya1* and *Six1* as its expression is markedly reduced in *Six1*-knockout mice, whereas *Eya1* and *Six1* expression is preserved in the absence of *Pax2* (Xu et al., 2003). In contrast, *Pax2* expression is unaffected in the otic epithelium of *Eya1^−/−^* or *Six1^−/−^* mice and vice versa (Xu et al., 1999; Zheng et al., 2003). Nevertheless, in both organs – as in the fly – this circuit controls proliferation and maintenance of a pool of progenitor cells, as well as signalling pathways that mediate cell-cell interactions essential for normal cell fate specification and morphogenesis. Although SIX1 has many different targets in ear sensory epithelia and in nephron progenitors, some are common (Li et al., 2020; O'Brien et al., 2016). The latter include *EYA1*/*EYA4*, *SIX1*, *GATA3*, cell cycle regulators and signalling pathway components, including FGFRs (Fig. 3). Thus, common target genes might implement similar processes in both organs, whereas context-specific interactors and co-factors define unique properties.

## The zinc finger transcription factor GATA3

In humans, haploinsufficiency or mutations in *GATA3*, encoding a dual zinc finger transcription factor, cause the autosomal dominant disease known as hypoparathyroidism, deafness and renal dysplasia (HDR) syndrome or Barakat syndrome (Bilous et al., 1992; Van Esch et al., 2000). Different *GATA3* mutations have been reported, including nonsense, frameshift and missense mutations, as well as intragenic deletions and mutations affecting splicing (Nesbit et al., 2004), with a recent study reporting a comprehensive literature review of phenotype-genotype relationships (Tao et al., 2023). HDR syndrome is a rare developmental disorder with variable clinical expression and age of onset. Sensorineural hearing loss (Box 2) is usually bilateral and may range from mild to profound. The renal disease includes nephrotic syndrome, cystic kidney, renal dysplasia, hypoplasia or aplasia, pelvicocalyceal deformity, vesicoureteral reflux, chronic renal failure, haematuria, proteinuria and renal scarring. Although hearing loss and hypoparathyroidism are the most prominent features in patients with HDR syndrome, renal defects are observed in 74% of patients, whereas 1.8-8% of patients present with seizures, apnea, cataract of patients, whereas intellectual disabilities (Tao et al., 2023).

*Gata3* is a key regulator of both auditory and renal development (Grote et al., 2006; Lawoko-Kerali et al., 2002). In the mouse ear, *Gata3* is initially expressed throughout the otic placode, with its expression becoming restricted laterally at E9.5-E10 (Lawoko-Kerali et al., 2002). At E11.5-E13.5, *Gata3* expression is high in the cochlea but is downregulated in the vestibular region (Karis et al., 2001; Lawoko-Kerali et al., 2002). *Gata3*-deficient mice display sensorineural hearing phenotypes, similar to those in patients with HDR syndrome (Grigorieva et al., 2010; Van Der Wees et al., 2004). In *Gata3*-null mice, the otic placode invaginates abnormally due to altered expression of adhesion molecules and decreased cell proliferation (Lilleväli et al., 2006). In addition, the cochlea fails to spiral and the canals do not form (Karis et al., 2001). When *Gata3* is conditionally deleted in otic cells at E8.5-E10.5, the prosensory domain is not specified and cell death is observed in the cochlear duct. As a result, the cochlea is disorganized and shortened, with no or significantly fewer HCs and SCs (Duncan and Fritzsch, 2013; Duncan et al., 2011; Luo et al., 2013). In chicks and mice, *Gata3* is downstream of *Six1* and *Pax2* (Christophorou et al., 2010; Zheng et al., 2003), and controls the specification and development of the prosensory domain by regulating the expression of *Pou4f3* (encoding a transcription factor) and *Cdkn1b* (encoding the cyclin-dependent kinase inhibitor p27^Kip1^) (Duncan and Fritzsch, 2013; Milo et al., 2009; Walters et al., 2017).

*Gata3* also plays a role in the maintenance, differentiation and maturation of neurons and HCs (Fig. 3). In *Gata3-*deficient mice, spiral ganglion neurons die around E18.5 owing to increased apoptosis (Luo et al., 2013). Furthermore, axonal navigation is severely disrupted (Karis et al., 2001), auditory synaptogenesis is reduced, and projections grow out prematurely towards the cochlear duct (Appler et al., 2013; Yu et al., 2013). Consequently, functional maturation of IHCs and their innervation is compromised (Bardhan et al., 2019). In HCs, *Gata3* expression is downregulated as they differentiate (Rivolta and Holley, 1998), suggesting that its expression is tightly regulated for HCs to form.

Finally, *Gata3* may regulate otic development by controlling FGF signalling by binding to the *Fgf10* enhancer (Economou et al., 2013). Inactivation of *Gata3* leads to a loss of *Fgf10* expression in otic epithelium and auditory ganglion, whereas *Fgf3* expression is not affected (Lilleväli et al., 2006). Accordingly, *Fgf10*-mutant mice show similar defects in vestibular sensory neurons and HCs as those in *Gata3*-deficient mice (Pauley et al., 2003).

During renal development, *Gata3* is expressed in the nephric duct from the time of its emergence in the intermediate mesoderm (gestation week 4 in humans or E8.5 in mice). Its expression appears to be regulated by *Pax2*/*Pax8*, reminiscent of early inner ear development (Boualia et al., 2013; Grote et al., 2006). *Gata3* is essential for the formation of the nephric duct and ureteric bud (Debacker et al., 1999; Labastie et al., 1995). In mice, its inactivation results in ectopic formation and aberrant guidance of the nephric duct, accompanied by enhanced cell division, in contrast to its role in ear morphogenesis, during which GATA3 promotes proliferation (Grote et al., 2006). Specific *Gata3* inactivation in the mouse metanephric duct leads to ectopic ureteric budding due to the premature differentiation of nephric duct cells, as well as the loss of *Ret* expression, leading to a spectrum of urogenital malformations (Grote et al., 2008). Molecularly, *Gata3* directly regulates *Ret* expression by physically binding to its enhancer region (Boualia et al., 2013). Finally, *Gata3* is also expressed in stromal progenitor cells and plays a critical role in the glomerular development (Grigorieva et al., 2019).

In summary, *Gata3* has multiple functions in different cell types of the developing ear and kidney. It regulates early otic morphogenesis, specification of the neurosensory and prosensory domain, HC differentiation and maturation, spiral ganglion neuron survival, as well as axon outgrowth and synapse formation. In the kidney, it controls pronephric and mesonephric morphogenesis and ureteric budding in the metanephros. In humans, *GATA3* mutations lead to HDR syndrome with variable phenotypes, likely resulting from developmental defects similar to those observed in mouse mutants. Impairment of neuronal and/or HC differentiation leads to sensorineural hearing loss, whereas failure of ureteric budding impairs not only formation of the renal collecting duct but also normal differentiation of the surrounding mesenchyme into nephrons. Despite early-onset hearing loss and biochemical marker testing, phenotypic variability and differences in penetrance make early diagnosis challenging. Given the prominence of hearing loss and hypoparathyroidism, also assessing renal defects at neonatal stages should be informative for diagnosis, as might next-generation sequencing to ascertain mutations in *GATA3*.

## The zinc finger transcription factor SALL1

The Sal family member *Sall1* is the vertebrate homolog of the *Drosophila* region-specific homeotic gene *spalt* (*salm*), a regulator of sensory organ development in flies (Buck et al., 2000; De Celis et al., 1999; Kohlhase et al., 1996; Osafune et al., 2002). In humans, *SALL1* is located on chromosome 16q12.1 and mutations lead to Townes–Brocks syndrome (TBS), an autosomal dominant disease that has some phenotypic overlap with BOR syndrome. TBS is characterized by external ear malformations with sensorineural hearing loss, thumb anomalies and anorectal and renal malformations (Engels et al., 2000; Kohlhase et al., 1998; Kiefer et al., 2003). The clinical presentation of TBS is highly variable. The incidence of kidney abnormalities, including renal dysplasia or agenesis, ranges from 20.5 to 62.5%, whereas the incidence of sensorineural hearing loss is between 29.5 and 87.5% (Kohlhase et al., 1999; O'Callaghan and Young, 1990). Although *Sall1*-null mice do not fully mimic the human phenotype (Nishinakamura et al., 2001), mice heterozygous for a mutant allele found in patients with TBS, *Sall1-ΔZn*, do (Kiefer et al., 2003). This mutation results in a truncated SALL1 protein, which acts as a dominant-negative form leading to de-repression of SALL1 targets (Kiefer et al., 2008, 2003). These mice display high-frequency sensorineural hearing loss, renal cystic hypoplasia and bone abnormalities in the wrist (Kiefer et al., 2003). This raises the possibility that in addition to *SALL1* haploinsufficiency, TBS may also result from mutations generating dominant-negative forms of SALL1 (Kiefer et al., 2002, 2003, 2008; Yang et al., 2020).

In the inner ear, *SALL1* is expressed in the otic placode in chicks (Chen et al., 2017). In mice, *Sall1* is expressed in the future endolymphatic duct at E10.5 (Zou et al., 2006) and in the prosensory domain at E14.5 (Yang et al., 2020). In the chick otic placode, *SALL1* acts as a transcriptional repressor of *FOXI3* (Anwar et al., 2017; Tambalo et al., 2020), possibly by association with the nucleosome remodelling and deacetylase (NuRD) complex (Box 2) (Kiefer et al., 2002; Lauberth et al., 2007; Lauberth and Rauchman, 2006). *Foxi3* itself is a critical regulator of early ear development and may prime cells to respond to otic placode-inducing signals (Birol et al., 2016; Thawani et al., 2023). In humans, FOXI3 is associated with craniofacial microsomia (Box 2) (Mao et al., 2023). As the placode differentiates, *Foxi3* expression diminishes, potentially due to SALL1 activity. It is therefore possible that *Sall1* truncation or loss leads to misregulation of *Foxi3* and consequently to ear malformations. *Sall1* also plays a role in the prosensory domain. In mice, deletion of one *Sall1* allele or the introduction of *Sall1-ΔZn* decreases the numbers of outer HCs (OHCs) at E18.5 (Yang et al., 2020). These observations suggest that SALL1 is involved in HC formation; however, the precise mechanisms are poorly understood.

During renal development, *Sall1* is continuously expressed in the metanephric mesenchyme and in early differentiating nephrons (Buck et al., 2001; Nishinakamura et al., 2001). Deletion of *Sall1* in mice results in renal agenesis or severe dysgenesis. In *Sall1-*mutant mice, ureteric bud outgrowth into the metanephric mesenchyme is incomplete, leading to failure of tubule formation and increased apoptosis in the mesenchyme (Nishinakamura et al., 2001; Nishinakamura and Takasato, 2005). In turn, failure of ureteric bud invasion leads to downregulation of the mesenchymal signals, *Gdnf*, *Bmp7* and *Wnt4*.

Like *Pax2* and *Six2*, *Sall1* is downstream of *Six1* in the metanephric mesenchyme (Fig. 3); its expression is reduced in *Six1*-null mice at E10.5 (Xu et al., 2003). Indeed, EYA1 and SIX1 proteins cooperate to activate *Sall1* directly by binding to its promoter (Chai et al., 2006). During nephrogenesis, *Sall1* is expressed in both nephron and stromal progenitors (Abedin et al., 2011; Ohmori et al., 2015), where it appears to have different functions. In nephron progenitors, it maintains stemness by restraining their differentiation into renal vesicles (Basta et al., 2014). It is thought to activate progenitor-related genes in *Six2*-positive nephron progenitors either indirectly or through direct binding to their enhancers. Similar to *Six2* loss of function, *Sall1* deletion in *Six2*-positive nephron progenitors results in progenitor depletion, ectopic renal vesicle formation and apoptosis of differentiating nephrons (Kanda et al., 2014; Self et al., 2006). In *Six2*-negative nascent nephrons, SALL1 acts as a repressor of differentiation (Kanda et al., 2014), likely through association with the NuRD complex, reminiscent of its action in the otic placode.

In stromal progenitors, *Sall1* restricts the expansion of nephron progenitors non-cell-autonomously (Ohmori et al., 2015). Deletion of *Sall1* in stromal progenitors leads to a reduction in decorin (DCN) (Box 2) expression, which then inhibits BMP-mediated nephron differentiation, and an increase in FAT4 (Box 2) expression, which activates the Hippo pathway (Box 2). Consequently, the pool of nephron progenitors expands. These observations suggest that *Sall1* regulates genes expressed in the metanephric mesenchyme that signal to the ureteric bud, cap mesenchyme and stromal cells, coordinating ureteric bud branching and nephron induction.

In summary, *Sall1* appears to act as a transcriptional repressor in the otic placode, while promoting OHC formation through unknown mechanisms. In the kidney, it acts as both activator and repressor to balance self-renewal and differentiation of nephron progenitors and to coordinate reciprocal signalling between the metanephric mesenchyme and ureteric bud. Thus, SALL1 plays a role in early developmental stages when cell fates are established and signalling events are crucial for the coordinated formation of complex structures. Maintaining progenitor populations is critical during kidney development, as the cap mesenchyme generates nephron progenitors over a prolonged period. Thus, failure of these early developmental processes is likely to cause the phenotypes observed in humans presenting with TBS.

## The FGF pathway

FGFs comprise a family of signalling molecules with 23 members identified to date. Mutations in *FGF8* and the genes encoding the FGF receptors FGFR1 and FGFR2 cause human oto-renal syndromes (Table 2). Activating mutations of *FGFR2* lead to Apert syndrome, Pfeiffer syndrome, Antley–Bixler syndrome and Beare–Stevenson syndrome, which are associated with hydroureter, unilateral renal aplasia (Box 2) and/or vesicoureteral reflux, with conductive hearing loss (Box 2) (Agochukwu et al., 2014; Walker et al., 2016). These rare syndromes often share other phenotypes, including craniosynostosis (Box 2), other craniofacial anomalies, skeletal defects, distal defects (i.e. of the fingers and toes) and developmental delay. In up to 95% of patients, Apert syndrome is due to a mutation in *FGFR2*. Unilateral renal agenesis is observed in newborns (Urdaneta-Carruyo et al., 2014), and the main cause of congenital hearing loss remains to be clarified (Rajenderkumar et al., 2005). Pfeiffer syndrome is an autosomal dominant condition associated with mutations in both *FGFR2* and *FGFR1*. The phenotypes are cranial (i.e. low-set ears and external auditory canal stenosis), cardiac and renal, including hydronephrosis and pelvic kidney (Amiji et al., 2020). Antley–Bixler syndrome is a rare form of syndromic craniosynostosis with additional systemic synostosis (Box 2), including radio-humeral or radio-ulnar synostosis. Some patients have congenital renal anomalies (Ko, 2016), including horseshoe kidneys, renal agenesis (Ghazle and Newcomb, 2015) and narrow ear canals (Robinson et al., 1982), which lead to profound bilateral conductive hearing loss. Genetic studies showed that mutations in *FGFR1* or *FGF8* (encoding the ligand for FGFR1) cause Kallmann syndrome (Dodé et al., 2007, 2003; Falardeau et al., 2008; Sato et al., 2004) (Table 2). Kallmann syndrome is a clinically and genetically heterogeneous disease involving gonadotropin-releasing hormone deficiency and olfactory bulb hypoplasia (Dodé and Hardelin, 2009; Kallmann, 1944; Oliveira et al., 2001). There are two forms of Kallmann syndrome: Kallmann syndrome 1 (KAL1) is X-chromosome linked and Kallmann syndrome 2 (KAL) is autosomal dominant. Various mutations in *FGFR1* or *FGF8* underlie the latter. Whereas unilateral renal agenesis has been found in approximately 30% of patients with KAL1 (Kirk et al., 1994), so far it has not been reported in patients with *FGFR1* or *FGF8* mutations. These findings suggest functional redundancy of different FGFs, as multiple FGFs and their receptors are expressed in the kidney (Cancilla et al., 2001, 1999). In contrast, hearing impairment is common to both forms of Kallmann syndrome (Dodé et al., 2007, 2003; Falardeau et al., 2008; Sato et al., 2004).

The FGF signalling pathway is repeatedly required during ear and renal development (Bates, 2011; Dorey and Amaya, 2010; Ebeid and Huh, 2017; Ladher et al., 2010; Walker et al., 2016). FGF signalling molecules regulate cellular proliferation, differentiation and migration by activating their tyrosine kinase receptors (FGFR1-4) (reviewed in Imamura, 2014; Ornitz, 2000). As mutations in *FGF8*, *FGFR1* and *FGFR2* are linked to human oto-renal syndromes (Table 2), we will focus on these factors and some of their relevant ligands (for in-depth reviews, see Bates, 2011; Riley, 2021; Walker et al., 2016). FGF8, FGF9 and FGF20 are known to bind to FGFR1, whereas FGF3, FGF7, FGF9 and FGF10 bind FGFR2.

At the start of ear development, different FGFs are expressed in the cranial mesoderm and in the hindbrain to mediate the induction of otic progenitors (Schimmang, 2007). *Fgf10* and *fgf3* are expressed in the mesoderm of mouse (Pirvola et al., 2000; Wright and Mansour, 2003) and zebrafish (Phillips et al., 2001), respectively; *Fgf3* is expressed in the mouse, chick and *Xenopus* hindbrain (Lombardo et al., 1998; Mahmood et al., 1995, 1996; Olaya-Sánchez et al., 2017; Tannahill et al., 1992); and *fgf8* is expressed in the zebrafish hindbrain (Phillips et al., 2001). In mice, *Fgf3* and *Fgf10* are both required for otic placode induction, as loss of either Fgf gene (Mansour, 1994; Ohuchi et al., 2000; Represa et al., 1991) leads to reduced *Pax2* expression and smaller otocysts, whereas otocyst formation is absent in *Fgf3*/*Fgf10* double-knockout mice (Alvarez et al., 2003; Léger and Brand, 2002; Wright and Mansour, 2003; Zelarayan et al., 2007). In contrast, ectopic expression of *Fgf3* or *Fgf10* induces *Pax2* expression and/or ectopic otocysts in the surface ectoderm or the developing hindbrain (Alvarez et al., 2003; Kil et al., 2005; Ladher et al., 2000; Phillips et al., 2001; Vendrell et al., 2000). Thus, the FGF pathway is critical for the initiation of inner ear development.

FGFs continue to play a role in otocyst patterning and morphogenesis (Adamska et al., 2001; McKay et al., 1996). *Fgf10* and *Fgf3* are expressed in the ventral and ventrolateral otocyst, respectively (Hatch et al., 2007; Pirvola et al., 2000). *Fgfr2*, encoding the FGF10/FGF3 receptor (Ornitz et al., 1996; Mathieu et al., 1995), is expressed in a complementary pattern in the non-sensory epithelium of the dorsal otocyst (Pirvola et al., 2000). In *Fgf3*-mutant mice, dorsal gene expression is reduced, leading to endolymphatic duct and membranous labyrinth malformations (Hatch et al., 2007; Mansour et al., 1993). In *Fgf10-*null mice, the ear is smaller with complete absence of the posterior canal system (Pauley et al., 2003). Likewise, deletion of an *Fgfr2* isoform results in severe dysgenesis of the cochleovestibular membranous labyrinth, similar to the phenotype of *Fgf3*/*Fgf10* double-mutant mice (Alvarez et al., 2003). Taken together, these findings show that FGF signalling is critical for patterning the otocyst and the formation of structures derived from the dorsal otocyst. In addition, *Fgf3* and *Fgf10* are also involved in sensory organ and ganglion development. In *Fgf3*-mutant mice, the posterior sensory domain and the cochlear-vestibular ganglion are smaller (Hatch et al., 2007), and a similar phenotype is observed in *Fgf10*-mutant mice (Pauley et al., 2003).

*Fgf9* and *Fgf20* control the outgrowth of the cochlea from the otocyst (Huh et al., 2015)*.* They are expressed in the non-sensory and sensory epithelium of the otocyst, respectively, whereas their receptor genes *Fgfr1* and *Fgfr2* are expressed in the surrounding mesenchyme. Deletion of *Fgf9* and/or *Fgf20* reduces cochlear epithelial cell proliferation and the size of the prosensory domain, thereby regulating the overall size of the cochlea (Huh et al., 2015; Pirvola et al., 2004). These findings suggest that in response to FGF, the mesenchyme activates signalling pathways to control cell proliferation in the developing cochlea.

In the cochlear sensory epithelium, IHCs, OHCs and specialised SCs are specified at later stages, with *Fgf8* and *Fgf20* playing an important role. At E16, *Fgf8* is expressed in IHCs, whereas its receptor gene *Fgfr3* is expressed in adjacent progenitor cells, which will ultimately develop as OHCs and the various supporting cells, namely, pillar cells, Hensen's cells and Deiters' cells (Box 2) (Jacques et al., 2007; Shim et al., 2005; Mueller et al., 2002; Pirvola et al., 2002). *Fgf8* deletion leads to fewer and smaller pillar cells, whereas *Fgf8* overexpression or activation of *Fgfr3* induces ectopic pillar cells at the expense of OHCs (Jacques et al., 2007). Thus, FGF8 induces pillar cells, while inhibiting OHC formation. In contrast, signalling through FGFR1, FGF20 promotes the differentiation of OHCs and SCs in the lateral compartment sensory epithelium (Hayashi et al., 2008; Ono et al., 2014; Pirvola et al., 2002). At E13.5, *Fgf20* is expressed in the future cochlear sensory epithelium and its deletion at E14 leads to a reduction of OHCs and SCs (Hayashi et al., 2008; Huh et al., 2012).

In the mouse kidney, *Fgfr1* and *Fgfr2* are expressed in both the metanephric mesenchyme and the ureteric bud from E10.5 onwards (Poladia et al., 2006). Conditional targeting approaches were used to determine their roles in specific cell types of the developing kidney. Mutant mice with compromised or lost *Fgfr2* function show unilateral renal agenesis, similar to patients with Apert syndrome (Urdaneta-Carruyo et al., 2014). *Fgfr2*, but not *Fgfr1*, is crucial for nephric duct and ureteric bud morphogenesis. *Fgfr2* deletion in the E11.5 nephric duct leads to regression of the caudal duct, aberrant ureteric bud branching, thin ureteric bud stalks and fewer ureteric bud tips due to increased apoptosis and reduced proliferation (Okazawa et al., 2015; Zhao et al., 2004). This is accompanied by non-autonomous defects in the surrounding mesenchyme, including thickened renal cortical stroma and fewer cap mesenchyme cells, resulting in fewer mature nephrons due to increased apoptosis from E13.5 onwards. Postnatally, renal hypoplasia leads to chronic kidney disease, hypertension and left ventricular hypertrophy (Poladia et al., 2006), reminiscent of renal symptoms in human syndromes. Ultimately, the adult kidneys are small and abnormally shaped or are hydronephrotic (Box 2).

During the formation of the metanephric mesenchyme, both *Fgfr1* and *Fgfr2* appear to work redundantly. When either receptor is deleted in the metanephric mesenchyme using a *Pax3*-*Cre* line, the kidney appears normal. However, double knockouts display renal aplasia with the metanephric mesenchyme almost absent and the ureteric buds remaining unbranched due to reduced proliferation and increased cell death (Poladia et al., 2006; Sims-Lucas et al., 2012). Mutant mesenchyme cells near the ureteric buds express *Eya1* and *Six1*, but do not express *Six2*, *Sall1* or *Pax2*, suggesting that FGF signalling regulates their expression (Poladia et al., 2006) and, consequently, the maintenance of nephron progenitors (Brown et al., 2011). Knockout of both *Fgfr1* and *Fgfr2* in *Six2-*positive progenitor cells leads to increased apoptosis, loss of stemness and ultimately renal cystic dysplasia (Di Giovanni et al., 2015)*.* Interestingly, this phenotype is mimicked by the loss of *Fgf9* and *Fgf20* (Barak et al., 2012), suggesting that FGF9 and FGF20 may be the relevant ligands that maintain the nephron progenitors, similar to their role in inner ear sensory progenitors. Because *Fgf9* is mostly expressed in the ureteric bud and *Fgf20* is expressed exclusively in nephron progenitors, it is likely that *Fgf9* and *Fgf20* signal to mesenchymal *Fgfr1*/*Fgfr2* in both a paracrine and an autocrine manner.

Finally, *Fgf8* is essential for gene regulation and cell survival at distinct stages of nephrogenesis, including the maintenance of nephron progenitors (Grieshammer et al., 2005; Perantoni et al., 2005). *Fgf8* is expressed in the metanephric mesenchyme surrounding the emerging ureteric bud at E12. From E12.5, the expression is resolved into discrete spots near the periphery of the metanephric mesenchyme and later the nascent nephrons (Grieshammer et al., 2005). Inactivation of *Fgf8* in mouse metanephric mesenchyme leads to nephron progenitor depletion and small kidneys, with a complete block in nephrogenesis past the renal vesicle stage.

In summary, FGF signalling has multiple roles at different stages of development of the inner ear and kidney, regulating cell proliferation, specification and differentiation. In humans, FGF8 is unique among FGF ligands as it is the only ligand for which mutations are associated with both renal and ear defects. All other ligands are likely to play redundant roles in both organs. In contrast, mutations in receptor genes, including *FGFR1* and *FGFR2*, have been linked to multiple syndromes associated with renal and inner ear phenotypes (Table 2). Patients diagnosed with these syndromes present with complex craniosynostosis phenotypes (Box 2) due to early closure of the cranial sutures. It is, therefore, challenging to distinguish primary and secondary effects of mutations in FGF pathway members. Given the pleiotropic role of FGF signalling, sensorineural hearing loss in patients is likely due to early developmental defects, including the outgrowth of the cochlear duct and specification of HCs and SCs in the cochlea. Likewise, in kidney development, specification of progenitors for specialised cell types, in the correct proportions and at the right time, is critical to establish a functional organ. In both organs, FGF crosstalk is critical to coordinate cell proliferation and differentiation across different cell populations, which, in turn, is necessary to build complex organs.

## Conclusions and perspectives

Over the last few decades, much progress has been made to define the molecular mechanisms controlling ear and kidney development and to identify genetic mutations underlying congenital malformations affecting the formation of both organs. Most transcription factors discussed here control the balance between proliferation, self-renewal and differentiation, ensuring that the right cell types, in the correct proportions, are generated. Other transcription factors also regulate the signalling crosstalk between different cell populations. Both are required for the morphogenesis of complex organs. However, direct targets of these factors have only been characterised in a few cell types, and the mechanisms by which the same factor controls different processes in different contexts are only beginning to be elucidated. Characterisation of the molecular pathways downstream of these transcription factors will not only provide insight into their mechanism of action, but also highlight the mechanistic similarities and differences in diverse cellular contexts and organs. This will require systematic experiments using state-of-the-art molecular profiling, as well as the identification of tissue-specific regulatory elements and their target genes. The latter will also provide a rich resource to discover previously unreported mutations in non-coding regions affecting normal ear and kidney development.

Recent development of single-cell technologies, such as RNA-sequencing and assay for transposase-accessible chromatin sequencing (ATAC-seq) techniques, have allowed us to explore the molecular makeup of individual cells at unprecedented resolution. Transcriptional and epigenomic profiling of the developing kidney has been reported in mice and human tissues and organoids (Combes et al., 2019b; Liao et al., 2020; Lindstrom et al., 2018; Miao et al., 2021; O'Brien et al., 2018, 2016; Wu et al., 2018), whereas less work has been performed on inner ear development (Buzzi et al., 2022; Grandi et al., 2020; Kolla et al., 2020; Sun et al., 2018; van der Valk et al., 2023). This technology has been transformative, allowing the identification of previously unreported cell types and dynamic changes of gene expression, as well as the inference of developmental trajectories and the prediction of regulatory regions and transcription factor targets. Bioinformatics tools integrating such information permit the construction of gene-regulatory networks that model how complex organs form and can predict consequences of genetic mutations. Recently, single-cell RNA sequencing has been applied to phenotyping mouse embryos carrying mutations for developmental disorders, allowing the identification of shared and common features (Huang et al., 2023). In the future, similar approaches in the context of oto-renal disease will help to disentangle complex developmental processes and how mutations in different genes may result in similar or distinct phenotypes. In turn, this will lead to a deeper understanding of the pathogenesis of oto-renal syndromes and ultimately help to establish better diagnosis and treatments.

Although much of our understanding on ear and kidney development comes from animal models, the molecular dissection of human organogenesis continues to be challenging. Aiming to recapitulate normal development, three-dimensional organoid cultures from human stem cells offer the unique opportunity to overcome such challenges and to examine the cellular and molecular phenotypes resulting from mutations found in patients. Kidney organoids are relatively well established (Clevers, 2016; Gupta et al., 2022; Takasato and Little, 2015), but organoids for modelling ear formation are in their infancy (Connolly and Gonzalez-Cordero, 2022; Nist-Lund et al., 2022; Roccio and Edge, 2019; van der Valk et al., 2021). Combined with transcriptional and epigenomic profiling, as well as state-of-the-art imaging approaches, organoids will help investigate how re-wiring of small regulatory circuits enable cells to develop organ-specific properties.

In addition, organoid systems are invaluable to model human disease (Tang et al., 2019; Tran et al., 2022). Organoids from patient-derived induced pluripotent stem cells or from genetically engineered stem cells offer the opportunity to investigate disease mechanisms, but also serve as a platform for drug discovery and development of personalised approaches for disease treatment. Combined with recent developments in gene editing tools, such as CRISPR/Cas9, organoids provide a powerful platform. Such technologies have also paved the way for modification of the human genome (Cong et al., 2013), with CRISPR-engineered cell therapies currently in clinical trials for cancer and immunological syndromes.

In summary, new technological advances in molecular and stem cell biology now provide powerful tools to investigate human disease. Their application to oto-renal syndromes will be instrumental to enhance mechanistic understanding and to develop new treatment options.
